# Direct Effect of Local Cryotherapy on Muscle Stimulation, Pain and Strength in Male Office Workers with Lateral Epicondylitis, Non-Randomized Clinical Trial Study

**DOI:** 10.3390/healthcare10050879

**Published:** 2022-05-10

**Authors:** Aleksandra Radecka, Anna Lubkowska

**Affiliations:** Department of Functional Diagnostics and Physical Medicine, Pomeranian Medical University in Szczecin, 71-210 Szczecin, Poland; aleksandra.radecka@pum.edu.pl

**Keywords:** surface electromyography, local cryotherapy, lateral epicondylitis, musculoskeletal pain, fatigue

## Abstract

Background: Local cryotherapy (LC) is one of the physiotherapeutic methods used in the conservative treatment of lateral epicondylitis (LE). The aim of the study was to verify the direct effect of a single LC procedure on the clinical symptoms of lateral epicondylitis enthesopathy (pain, pain free grip, PFG) and its effect on the bioelectrical properties of the wrist extensor muscles at rest, on maximal contraction and isometric contraction during fatigue. Methods: The study group was 28 men (35.4 ± 6.13 years) with confirmed unilateral epicondylitis. The performed procedures included the assessment of pain (visual analogue scale, VAS), PFG and A_RMS_ (root-mean-square amplitude) and mean frequencies (MNF) of the sEMG signal before (T_0_) and after (T_1_) LC on the side with enthesopathy (ECR_E_) and without enthesopathy (ECR_N/E_). Results: There was an increase in the ARMS values of the signals recorded during rest and MVC from the ECR muscles both with and without enthesopathy (*p* = 0.0001, *p* = 0.006), an increased PFG after LC only on the side with LE (*p* < 0.0001) and decreased pain (*p* < 0.0001). During isometric fatigue contraction, a higher ARMS on both the ECR_E_ side (*p* < 0.0001) and the ECR_N/E_ side (*p* < 0.0001) was observed after LC treatment, and a lower MNF was observed on both the ECR_N/E_ side (*p* < 0.0001) and the ECR_E_ side (*p* < 0.0001) after LC. Conclusions: LC reduces the pain and increases PFG and muscle excitation expressed by ARMS and seems to delay muscle fatigue.

## 1. Introduction

Tennis elbow, lateral epicondylitis, or lateral epicondylalgia (LE) is a clinical term for the pain syndrome connected with enthesopathy lesions in the common extensor tendon that is formed by the extensor carpi radialis brevis and longus, extensor digitorum, extensor digiti minimi and extensor carpi ulnaris muscles [1,2]. The etiology of LE is associated with recurrent microtraumas caused by repetitive movements such as wrist grip or extension, radial deviation and/or forearm supination [3]. Previously, it was thought that the symptoms of LE were associated with the inflammatory process, especially in its early stages, but histopathological examinations did not reveal the presence of inflammatory cells in chronic LE biopsies [4]. New evidence identifies LE as a tendinosis, a symptomatic degenerative process characterized by abundant fibroblasts, vascular hypertrophy, and unstructured collagen [3,5]. On microscopic imaging, the tendon is characterized by an increase in the number of immature type III collagen fibers, loss of collagen continuity, an increase in the amount of basic substance, and a chaotic increase in pathologic vascularization [5]. In situations of repetitive stretching, multiple micronephral tendons potentially cause irreversible denaturation of matrix proteins and proliferation of fibrous tissue [6]. Forming scar tissue is susceptible to injuries, and immature repair causes more serious tears and, in the chronic period, degenerative changes and failure of the musculotendinous biomechanics [3]. Pain in LE is of neurogenic etiology, as indicated by the presence of nerve fibers showing reactivity to neuropeptides, including substance P (SP) and calcitonin gene-related peptide (CGRP) [7,8,9]. The role of neurogenic inflammation is also supported by the fact that CGRP mRNA and IL-1α mRNA levels were inhibited after the administration of corticosteroids effective in the temporary relief of pain in LE [3].

The main clinical symptoms of LE include: pain or burning around the lateral epicondyle of the humerus, which frequently radiates down the forearm and sometimes extends proximally to the upper arm [3], pain usually triggered or exacerbated during: resisted wrist extension, resisted middle finger extension, and passive wrist flexion. Another symptoms are decreased grip strength, and tenderness on palpation over the facet of the lateral epicondyle [1,10,11]. An average period of an LE episode ranges between 6 months and 2 years. It affects most activities of daily living (ADL) and could lead to considerable functional disability and a loss of performance in occupational and sport activities [1,12,13].

There is a distinction between surgical and non-surgical methods of LE treatment, with non-surgical treatment remaining the priority and surgical interventions being recommended in difficult cases. Surgical methods include open surgery, percutaneous surgery, and arthroscopic surgery. Nonoperative methods, on the other hand, include activity modification, anti-inflammatory medications, counterforce braces, autologous blood injection, platelet-rich plasma (PRP) injection and physiotherapy [3]. Physiotherapy includes exercises (stretching exercises, eccentric epicondylar muscle strengthening exercises) [5], mobilisation, manipulation, deep transverse friction massage and different kinds of physical agents (shockwave, laser, low-frequency transcutaneous electrical nerve stimulation, ultrasound, pulsed magnetic wave therapies, kinesiology taping) [11,14,15,16,17] as well as local cryotherapy [5,18,19,20]. Local cryotherapy (LC) is a therapy in which a small area of the body is exposed to low temperatures [21]. The term local cryotherapy is often understood very broadly, covering the use of various cooling agents and various treatment procedures. Among the many forms of LC (ice [5], ice bag [22], gel packs [23], water immersion [24]), refrigerant gases (liquid nitrogen vapours [21], carbon dioxide microcrystals [25], cold air [26]) are most commonly used clinically. The history of using cold in medicine dates back to ancient times, when snow, ice and water mixtures and cold water were used [27,28]. Clinically, the most common applications of cryotherapy include the treatment of musculoskeletal disorders such as osteoarthritis [29,30], osteoporosis [31,32], rheumatoid arthritis, post-traumatic stress disorders, ankylosing spondylitis, spinal pain syndromes [32] and fibromyalgia [33]. Contraindications to cryotherapy are cold intolerance, Raynaud’s disease, open wounds and skin lesions, cold urticaria, and gangrenous lesions [32].

Cold therapy induces a number of physiological reactions such as analgesic effects, neuromuscular effects, and anti-inflammatory and antiedema effects [21]. Tendinosis begins with tendinitis, which then instigates a healing process that changes the collagen and weakens the tendon, becoming tendinosis [5]. The benefits to LE of lowering tissue temperature include the ability to reduce extravasation of blood and protein from new capillaries present in tendinopathy and to reduce the metabolic rate of the tendon, which promotes healing of LE [18,34]. Local decreases in tendon temperature causes vasoconstriction and is believed to inhibit the abnormal neovascularization of tendon tissue [5]. In addition, local cryotherapy can be used for symptomatic relief of pain [19]. It has also been shown that cooling of the skin could improve endurance exercise performance [35,36], and excessive external cooling could reduce muscular performance as a result of reductions in muscle/nerve conduction velocity [37]. However, it is not known how local cryotherapy may affect muscle performance during fatigue isometric contraction in enthesopathy.

Previously, studies have been conducted on the effect of various rehabilitation procedures, including LC, on clinical symptoms of LE (pain, grip strength) [18,38]. However, no studies have been found that assessed the effect of cryotherapy alone. This seems to be very important for the assessment of the validity of LC application in LE therapy.

Surface electromyography is a recommended research tool in sport and rehabilitation sciences because sEMG allows for non-invasive insight into how the neuromuscular system behaves (myoelectric features of the neuromuscular activation associated with muscle contraction) [39]. The sEMG apparatus is a highly sensitive voltmeter that detects increases and decreases in the muscle voltage resulting from depolarisations and hyperpolarisation of the muscle fibre membrane [40]. An electromyographic parameter that determines the value of mean voltage being generated by muscle MUs is amplitude (μV), while the parameter of signal density is the indicator of the frequency spectrum [40].

The most commonly used sEMG parameters are root-mean-square amplitude (ARMS), mean frequency (MNF) and median frequency (MDF) [41,42]. It has been well established that a decrease in MNF or MDF indicates a shift in sEMG from high to low frequencies, which is associated with the decreased conduction velocity of action potentials in fatigued muscles [42]. During sustained submaximal static contractions the signal amplitude (RMS) also increases. Both shifts in the EMG spectrum and amplitude (RMS) are used as markers of muscle fatigue [43,44,45,46]. The above-mentioned myoelectric manifestations of fatigue are explained by the slowing of motor-unit action potentials due to reduced conduction velocity and the synchronisation of motor units by the central nervous system to increase the mechanical output when the whole motor-unit pool is recruited [47].

Surface electromyography has been previously used to investigate the function of the forearm muscle in healthy and LE individuals. Based on the sEMG examination, the following was found: a muscular asymmetry characterised by decreased activation of the radial wrist extensor muscle, a compensatory increase in activation of the ulnar extensor muscle and a higher rate of muscle fatigue in LE subjects compared with controls [48,49,50].

Surface electromyography has also been used for assessing neuromuscular function after different types of exposure to cold, for example: cold water immersion [51,52], cold pack [53], partial-body cryotherapy [47,54], a computer-controlled cooling device that administered continuous cooling through a knee cuff [55], and ice massage [56]. Nevertheless, no studies have been found that assessed the activity of extensor muscles with enthesopathy subjected to cryotherapy.

The aim of the study was to verify the direct effect of a one-time local cryotherapy treatment with the use of cryogenic gas on the clinical symptoms of lateral epicondylitis (pain and pain-free grip) and its effect on the myoelectrical activity of extensor carpi muscles at rest on maximal contraction and isometric contraction during fatigue in male office workers.

## 2. Materials and Methods

### 2.1. Participants

**Figure 1:**
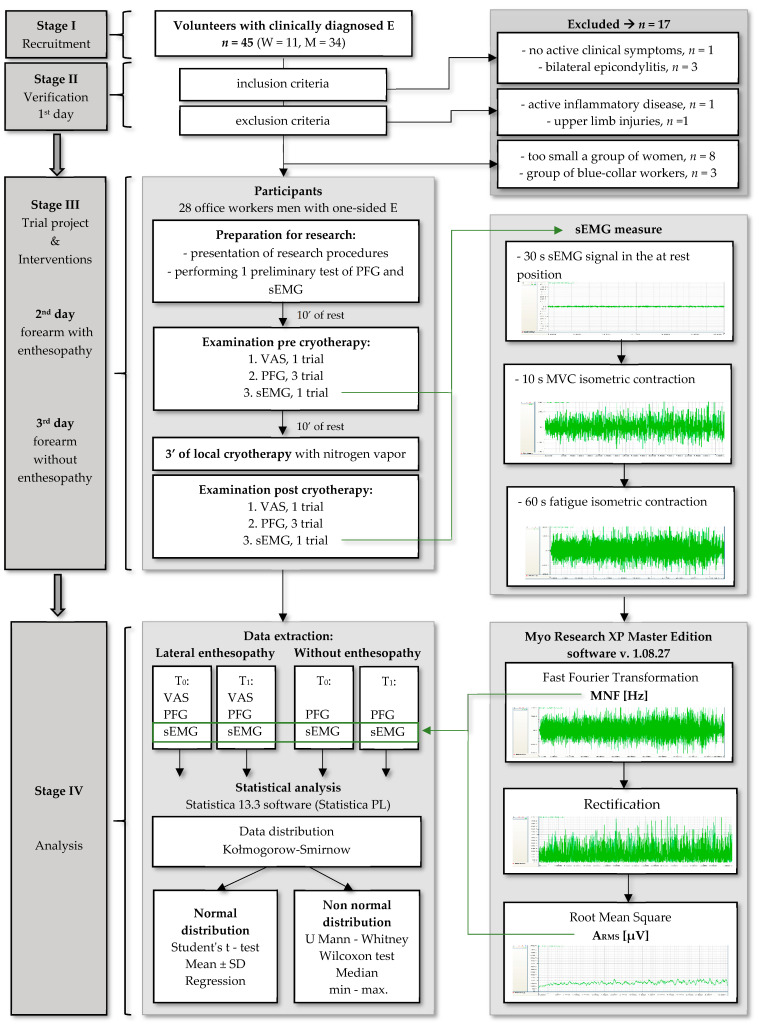
The flow chart presenting the developed research process.

The study was approved by the local Ethics Committee (Ref. KB-0012/36/13). Each of the subjects included in the study was informed in detail about examination procedures and signed a written informed consent according to the Declaration of Helsinki. In the first stage of the study, 45 volunteers were recruited who were clinically diagnosed with lateral epicondylalgia. In stage II, the participants were additionally subjected to medical verification (clinical examination) of confirmed LE. The subjects were also asked to complete a personal questionnaire, the results of which allowed for confirmation of the inclusion criteria of this research (Figure 1).

Criteria for inclusion were as follows:

- Age from 25 to 40 years;
- Pain around the lateral epicondyle during the extension of wrist and fingers against resistance and/or pain with resisted supination, pain with passive stretch to the wrist extensor or supinator muscles, and tenderness over the lateral epicondyle;
- Symptoms lasting for at least 3 months;
- Unilateral epicondylitis.

Subjects with systemic inflammatory disease, fibromyalgia syndrome, cervical radiculopathy, peripheral neuropathy in the upper extremity, identified neurological and myogenic diseases, history of upper limb injuries and hospitalisation in the last 6 months due to infectious diseases were excluded. Only 11 women entered the study, 8 of whom met the inclusion criteria and entered the study group. The group of women turned out to be too small for reliable statistical analyses (sample size). The combination of a group of women and men was considered inappropriate due to the significant influence of gender on strength, perception of pain and characteristics of the sEMG signal. Due to this, it was decided to completely exclude the group of women from the next stage of the study. In addition, 3 blue-collar workers were excluded from the study due to the different nature of their work, which could affect muscle strength and pain perception. Finally, the study involved 28 men with a mean age of 35.4 ± 6.13 years and a mean body mass index of 24.6 ± 4.01 kg/m^2^. The participants were office workers (12 programmers, 8 IT specialists, 5 statistical analysis specialists, and 3 corporate office workers). After being included in the study, the participants were instructed to refrain from any exercise and not to use any painkillers during the next stage III.

### 2.2. Experimental Design and Protocol

In the third stage of the study, all measurement procedures and cryotherapy treatments were performed on two consecutive research days. Examination procedures were performed on one side only (with or without enthesopathy). The order of the examined forearms was random. Measurements were carried out in the morning between 9:00 a.m. and 12:00 p.m. at an ambient temperature of 21–23 °C. Examination procedures were carried out in the following order:

1. Preparation for research
  - Detailed presentation of examination procedures by the researcher;
  - Skin preparation for sEMG measurement;
  - Making sure that procedures were understood and performing 1 PFG and sEMG test run;
2. After 10 min of rest, the subject assessed pain in the lateral epicondylar region on a 10-point VAS scale (visual analogue scale) felt during the resisted extension of the wrist in the upper limb diagnosed with LE;
3. Immediately after VAS, PFG was assessed;
4. Immediately after PFG, the extensor carpi radialis activity was assessed (EMG) during the rest position, and maximal voluntary contraction (MVC), and 60 s of fatigue isometric contraction were measured;
5. After 10 min of rest, a local cryotherapy treatment with liquid nitrogen vapours in the area of the lateral epicondyle and forearm was performed;
6. Immediately after the end of LC, the measurement procedures were repeated in the indicated sequence (points 2–5).

### 2.3. Pain Assessment

One of the most troublesome symptoms of LE is pain in the area of the lateral epicondyle, which occurs during the activity of wrist extensors. Therefore, to evaluate the clinical effect of LC, we assessed pain on the VAS scale during resisted wrist extension before and immediately after the treatment [18,38,57]. Pain was measured on a visual analogue scale (VAS), where 0 (cm) was “least pain imaginable” and 10 (cm) was “worst pain imaginable” [58]. The pain VAS was used to measure pain during resisted wrist extension of the forearm with lateral enthesopathy before and immediately after local cryotherapy.

### 2.4. Pain-Free Grip (PFG)

We also assessed pain-free grip strength because it is commonly measured to quantify the progression of LE. We decided to measure pain-free grip strength (PFG) instead of maximal handgrip strength (HGS) because PFG, apart from adequate interobserver reproducibility and advantageous interobserver reliability, shows a better correlation with common pain scales [50,59]. The PFG test was performed according to the measurement standards recommended for LE assessment, which include a standing position during the measurement and positioning the upper limb with a relaxed elbow in full extension along with the body. The subjects also received standardised instructions: they were to squeeze the dynamometer slowly until they began to feel discomfort [59]. The PFG was measured 3 times, with a 20-s rest interval between each measurement, respectively, for the LE limb and the non-LE limb. The results were averaged for better reproducibility. The PFG was assessed using a SAEHAN digital hand dynamometer (DHD-1 Digital Hand Dynamometer). The grip strength measurement range for DHD-1 is from 0 to 90 kg.

### 2.5. Surface Electromyography

Symptoms such as pain and decreased grip strength are both a cause and effect of abnormal muscle activity in LE. For this reason, we searched for an objective method to assess the activity of the wrist extensor muscles, and we assessed it both before and after LC exposure. To assess the muscle activity, surface electromyography (sEMG) was applied, which is recommended for the global characterisation of muscle activity in sport and rehabilitation sciences [60,61].

The bioelectrical activity of the ECR muscles was tested in a sitting position, starting with recording a 30-s electromyographic signal in the rest position. The rest position was characterised by wrist and palm support and flexion of the glenohumeral and humeroulnar joints of the examined limb (maintaining a position that was comfortable for the subject). Registration of the records during MVC and fatigue took place using the identical position as in the rest-related procedure, except for the absence of wrist and palm support. The subjects performed a 10 s and then 60 s isometric contraction against static resistance, but other examination procedures were kept unchanged (Figure 1).

During each session, two electromyograms were made simultaneously recording the signal from the extensor carpi radialis longus and brevis (ECR) muscles before and after local cryotherapy treatment (T0 and T1) on the region corresponding to the muscles being assessed.

The Surface EMG for Non-Invasive Assessment of Muscles (SENIAM) recommendations for scientific research using sEMG were included in the measurement. The skin over the muscle bellies under investigation was shaved and wiped with alcohol. EMG electrodes were placed on the extensor carpi radialis longus and brevis muscles of both forearms. The reference electrode (ground) was placed on the olecranon. For recording the signal, Noraxon Ag/AgCl dual electrodes (with a 1-cm diameter and an inter-electrode distance of 2 cm) were used [61,62].

A 4-channel Myotrace 400 Noraxon electromyograph was used for the tests.

Measurement of the muscle activity was performed according to the standard Amplitude Analysis and Frequency/Fatigue Analysis protocol available in Myo Research XP Master Edition software (v. 1.08.27). Recommendations for the assessment of MVC and fatigue isometric contraction were included. The subjects performed one maximal voluntary contraction (MVC) followed by a 60 s fatigue isometric contraction of 60–80% MVC. The MVC force measurement was performed using a KORDYNACJA digital push-pull PFD dynamometer, which was also used to maintain 60–80% MVC during fatigue isometric contraction by biofeedback (visualisation of the force value on the computer screen).

Mathematical analysis of electromyograms was also performed using Myo Research XP Master Edition software (Figure 1). To assess the density of bioelectrical signal, raw electromyograms were used, which were subjected to mathematical analysis with the fast Fourier transform (FFT) algorithm in order to determine the values of mean frequencies for amplitude changes. Determination of the objective values of amplitude was associated with the necessity of prior record rectification (straightening out) and creation of an envelope (smoothing) by applying the calculation algorithm RMS (root-mean-square) [61,63,64]. These analyses were made independently for each of the 1000 ms time intervals, which allowed for the determination of any possible changes in the parameters in the time domain, as well as for the whole electromyogram, in order to demonstrate a possible change in the mean values of parameters before and after exposure.

### 2.6. Local Cryotherapy

Local cryotherapy treatment was performed for 3 min with a local cryotherapy apparatus Kriopol R (Poland) using liquid nitrogen vapours (nozzle mouth temperature of −160 °C), directing the stream of gas perpendicularly to the surface of the dorsal forearm from the lateral epicondyle to the wrist. The nozzle distance from the surface of the cooled skin was 10 cm, while the exposure time was 3 min.

All subjects completed all examination and therapy procedures.

### 2.7. Statistical Analysis

Statistical analysis was performed using Statistica 13.3 software (Statistica PL, StatSoft). Data distribution was assessed using the Kolmogorov–Smirnov test. For the data with normal distribution, descriptive statistics were presented as the arithmetic mean and standard deviation. The data with a non-normal distribution were presented as the median and minimum and maximum values. For the data with normal distribution, the Student’s *t*-test for dependent variables was used to assess the effect of cryotherapy, and the Student’s *t*-test for independent variables was used to assess differences between the ECRE and ECRN/E groups. For the data with a non-normal distribution, the Mann–Whitney U test was used for intergroup comparisons (ECRN/E vs. ECRE), and for the assessment of the effect of local cryotherapy (T_0_ vs. T_1_), the Wilcoxon signed-rank test was used. Additionally, the sample size was calculated for the tests used, which, depending on the test and the analysed variable, ranged from 9 to 27. The ARMS and MDF values extracted from fatigue electromyograms from 28 subjects were averaged for each 1 s time window. Then, the linear regression equation was determined, and the results were presented in a scatter plot. Additionally, the accuracy of the estimated equation was assessed using multiple regression.

## 3. Results

### 3.1. Effect of Local Cryotherapy on Resting and MVC Signal Amplitude, PFG and VAS

**Figure 2:**
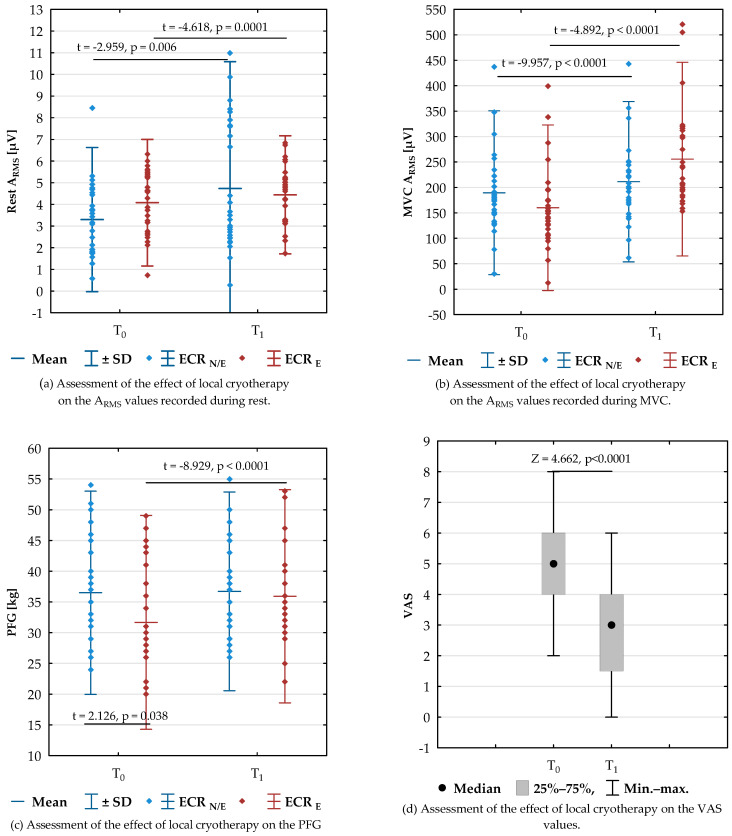
Assessment of the effect of local cryotherapy on the A_RMS_ values recorded during rest (**a**) and MVC (**b**) and on the PFG (**c**) and VAS values (**d**). Legend: ECR—extensor carpi radialis longus and brevis muscles, _LE_—lateral epicondylitis, _N/LE_—healthy side, A_RMS_—root-mean-square amplitude, MVC—maximum voluntary contraction, PFG—pain-free grip, VAS—visual analogue scale.

The analysis of the collected research material started with the evaluation of the effect of LC treatment on the resting value of the RMS amplitude for ECR_N/E_ and ECRE. There was a significant increase in the A_RMS_ values of signals recorded from the ECR muscles both with (*p* = 0.0001) and without enthesopathy (*p* = 0.006) (Figure 2a). Similarly, the RMS amplitude of the signal recorded from both ECR muscles during maximal isometric contraction was higher after LC (ECR_N/E_ *p* < 0.0001, ECR_E_ *p* < 0.0001) (Figure 2b). It is worth noting that the PFG value significantly increased after LC only on the side with enthesopathy (*p* < 0.0001) (Figure 2c). After LC, pain during resisted wrist extension decreased (*p* < 0.0001) (Figure 2d).

Except for the PFG values before therapy (ECR_N/E_ vs ECR_E_ *p* = 0.038), there were no significant differences in the parameters analysed (rest-A_RMS_, MVC A_RMS_, PFG) between the side with enthesopathy and without enthesopathy both before (T_0_) and immediately after (T_1_) local cryotherapy (Figure 2a–c).

### 3.2. Effect of Local Cryotherapy on Electromyographic Indices of Fatigue

**Figure 3:**
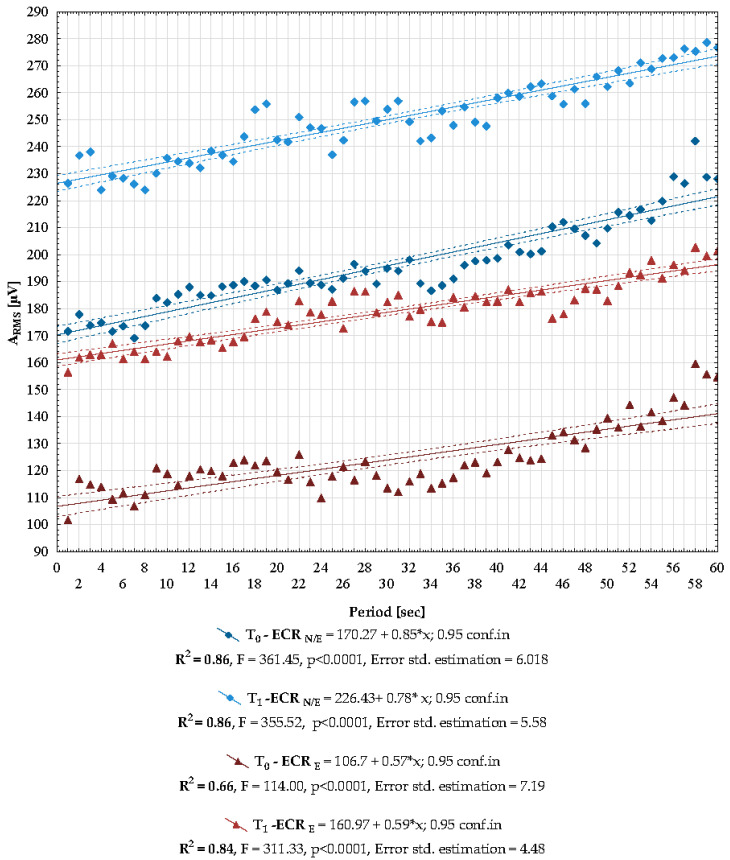
Change in the A_RMS_ value recording during a 60 s fatigue isometric contraction. Legend: A_RMS_—root-mean-square amplitude, ECR—extensor carpi radialis longus and brevis muscles, _E_—enthesopathy, _N/E_—healthy side.

**Figure 4:**
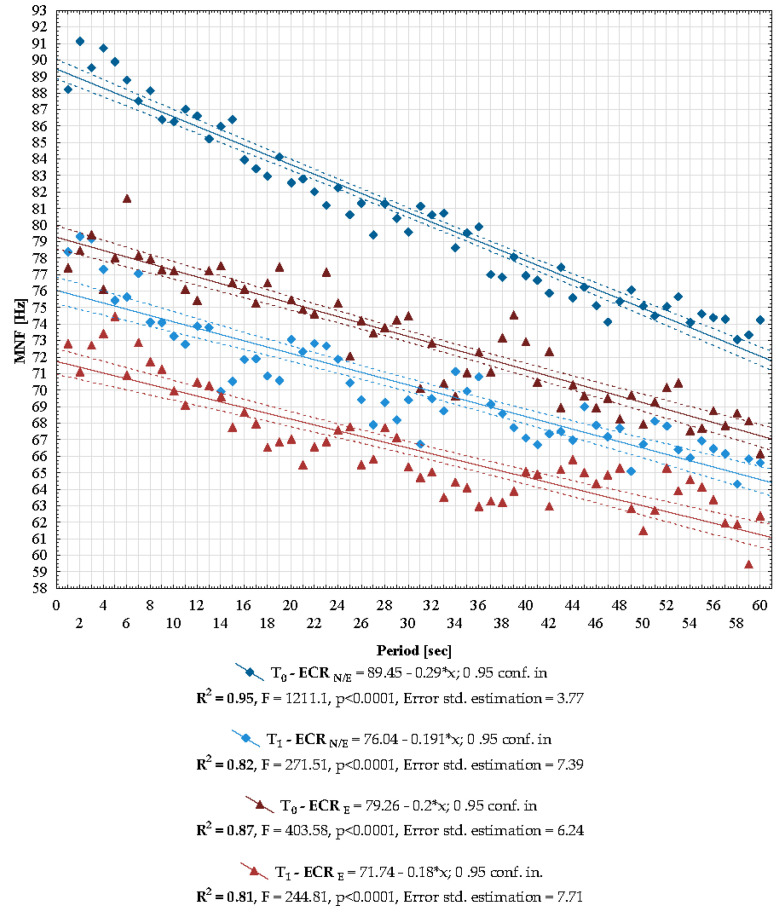
Change in the NF value recording during a 60 s fatigue isometric contraction. Legend: ECR—extensor carpi radialis longus and brevis muscles, _LE_—lateral enthesopathy, _N/LE_—healthy side, MNF—mean frequency.

The present study also analysed the direct effect of LC treatment on the electromyographic activity of the ECR muscles recorded during fatigue isometric contraction. As expected, an increase in the A_RMS_ values with a concomitant decrease in MNF was found during a 60 s contraction. The above-described characteristics of the electromyographic signs of fatigue were found in both electromyograms recorded from ECR_N/E_ and ECR_E_, before (T_0_) and after (T_1_) the LC therapy was applied. An increase in A_RMS_ and a decrease in MNF during 60 s of contraction are shown in detail in Figure 3 and Figure 4, respectively. The determined regression lines and the equations describing them (placed below the graphs) further confirm an increase in the A_RMS_ values and a decrease in MNF during contraction (Figure 3 and Figure 4). As in the resting recording and during the maximal contraction, a significantly higher value of the RMS amplitude was found after stimulation with LC (y-axis is always higher after LC treatment in ECR_N/E_: T_0_ = 170.27 vs. T_1_ = 226.43, ECR_E_: T_0_ = 106.7 vs. T_1_ = 160.97). The increased value of A_RMS_ after LC was maintained throughout a 60 s fatigue isometric contraction, while the values of the slope coefficients of the regression line were similar (ECR_N/E_: T_0_ = +0.85 vs. T_1_ = +0.78, ECR_E_: T_0_ = +0.57 vs. T_1_ = +0.59) (Figure 3). At the same time, an increase in the A_RMS_ value over time, described by the slope of the regression line, is significantly higher on the side without enthesopathy compared to the side with pathology (Figure 3). Except for the record recorded with ECR on the side with enthesopathy before the LC treatment was applied (ECR_E_ R_2_ = 0.66), the coefficients of determination for the presented regression models are high (T_0_ ECR_N/E_ R^2^ = 86, T_1_ ECR_N/E_ R+ = 86, T_1_ ECR_E_ R^2^ = 84), confirming the high reliability of the analysis (Figure 3).

Data on median A_RMS_ values from the entire 60 s recording are placed in Table 1. Significantly higher A_RMS_ values were found on the ECR_N/E_ side compared to ECR_E_ both before (*p* < 0.0001) and after LC (*p* < 0.0001). At the same time, an increase in A_RMS_ values was found on both the ECR_N/E_ side (*p* < 0.0001) and the ECR_E_ side (*p* < 0.0001) after LC treatment.

The MNF value decreased during fatigue isometric contraction. Interestingly, in contrast to A_RMS_, the intersection of the regression line for MNF with the y-axis was a little lower after LC (ECR_N/E_: T_0_ = 89.45 vs. T_1_ = 76.04, ECR_E_: T_0_ = 79.26 vs. T_1_ = 71.74), while a periodic decrease described by the slope coefficient of the regression line after LC was also similar (ECR_N/E_: T_0_ = −0.29 vs. T_1_ = −0.19, ECR_E_: T_0_ = −0.2 vs. T_1_ = −0.18) (Figure 4). All the presented regression models characterising changes in the MNF values during a 60 s contraction are highly reliable (T_0_-ECR _N/E_ R^2^ = 0.95, T_1_-ECR_N/E_ R^2^ = 0.82, T_0_-ECR_E_ R^2^ = 0.87, T_1_-ECR_E_ R^2^ = 81 (Figure 4).

The median MNF values from the fatigue records are placed in Table 2. Significantly higher MNF values were found on the ECR_N/E_ side compared to the ECR_E_ both before (*p* < 0.0001) and after LC (*p* < 0.0001). At the same time, after LC, MNF values were lower on both the ECR_N/E_ (*p* < 0.0001) and the ECR_E_ side (*p* < 0.0001).

## 4. Discussion

In the present study, we investigated the direct effect of a single LC procedure on characteristics of sEMG signals recorded during rest, MVC and isometric fatigue contraction in patients with lateral epicondylitis enthesopathy. We compared the effect of LC on the surface electromyographic signal recorded from both the radial wrist extensors on the side with enthesopathy and on the healthy side. In addition, we assessed the effect of a single LC procedure on pain experienced during resisted wrist extension and strength of the pain-free grip of the affected side.

Due to the positive effect of cold on pain relief, inhibition of inflammation, regulation of muscle tension and stimulation of blood circulation, cryotherapy is a frequent adjunct to basic therapy of persistent musculoskeletal pain [19,20,38]. Although LC is recommended as a supplement to an exercise programme in lateral elbow tendinopathy, only single studies have examined its effectiveness [16,18]. Therefore, the present study assessed the effect of LC on pain perception during the provocation test and PFG, which is impaired in lateral epicondylitis enthesopathy.

The present study confirmed a decreased PFG value on the lateral epicondylitis enthesopathy side, as reported in the literature. This seems to confirm the validity of PFG as a screening test for this enthesopathy (Figure 2c) [50]. Immediately after LC application, the PFG value increased only on the side with enthesopathy (Figure 2c). At the same time, there was a significant reduction in the pain experienced during resisted wrist extension immediately after LC (Figure 2d). In view of this, the recorded increase in strength was probably related to the effect of low-temperature analgesia. The analgesic effect is caused by microvasculature alterations that decrease the production of inflammatory mediators, decrease local edema, disrupt the overall inflammatory response, and reduce nerve conduction velocity. The analgesic effect of cryotherapy is well documented in the literature. Cooling the area has been proven to relieve pain and reduce pain medication intake in patients with postoperative pain for orthopedic reasons and in patients with injuries and degeneration of the tendons [65,66,67,68].

A similar effect of increased grip strength (pain-free grip or handgrip strength) and reduced pain was demonstrated after an exercise programme with ice therapy [18], after manual therapy and local cryostimulation (high-pressure refrigerated carbon dioxide microcrystals at −78 °C) [38], after exercise with a conventional cold pack [57], and after cryoultrasound therapy sessions (a new technology that combines the therapeutic ultrasound 1.8 W/cm^2^ with cryotherapy at a temperature of −2 °C) [69]. Although cryotherapy is one of the therapies that make up a comprehensive rehabilitation program for treating lateral elbow tendinopathy [70], no evidence was found evaluating only the effect of LC on this enthesopathy symptoms. In all the studies cited, LC is combined with other therapies, making it impossible to reliably assess its effect on the symptoms experienced. It seems that the presented study is the first to verify the direct effect of a typical local cryotherapy procedure on the clinical symptoms of LE.

In the presented study, the direct effect of LC on the myoelectric activity using the sEMG method was assessed. In recent years, there has been a dynamic increase in the use of sEMG as a technique to objectify muscle function assessment for the purpose of, among others, rehabilitation and sports medicine [39]. We also decided to use the sEMG method to assess whether cryotherapy affects muscle excitation during various activities and in people with enthesopathy.

The evaluation of the sEMG signal recorded during rest showed an increase in the A_RMS_ value after the single therapy was applied. The described increase occurred both on the side with and without LE (Figure 2 a,b). The normative bioelectric activity of the muscles at rest is characterised by a minimal electrical voltage. When recorded with sEMG, it reaches amplitude values that do not exceed 25 μV [71,72]. It has been found that the average value of the amplitude of resting electromyograms from carpal radial extensor muscles is 4.5 to 5.9 μV, in healthy people, without a significant change in the circadian rhythm [73]. In our study, the recorded myoelectric activity at rest was like the above-mentioned one, before local cooling, averaging 3–5 µV, and after surgery, significantly increasing to 9–10 μV. Despite the real increase, the amplitude did not exceed the normative values (Figure 2a). There were no significant differences in the A_RMS_ resting value between the enthesopathy side and the healthy side both before and after LC (Figure 2a). The above indicates that the increased muscle excitation expressed by an increase in the value of A_RMS_ occurs independently of enthesopathy in response to the stimulation of extremely low temperatures. Although the resting sEMG signals were used to objectively evaluate the muscular response to various sorts of manipulations [74], to assess the daily variability of wrist extensor muscle activation during forearm rest in healthy volunteers [73], we were unable to find studies that included LC with which we could compare the obtained results.

Additionally, the effect of a single exposure to LC on the maximum voluntary contraction, both with and without enthesopathy, has been observed (Figure 2b). During the voluntary contraction, the amplitude of the sEMG signal increases dramatically, which is the effect of an increase in the muscle excitation during contraction and muscle activation. The definition “muscle activation” refers to the state of a muscle and is related to the magnitude of the force that a muscle actively produces (i.e., not including passive contributions) in relation to its maximum ability to produce force actively. Activation does not consider muscle contraction dynamics (force-length, force-velocity, etc.). Therefore, activation is related to the number of fibres that are active and not the force-generating capacity of those fibres. Activation can be estimated from muscle excitation and sEMG, but the relationship is not straightforward. However, based on rectified sEMG amplitude (used in this study), we can scale the level of global muscle excitation during activity [39,75].

It has been found the mean A_RMS_ of 150–170 μV for the ECR was recorded during maximum voluntary extension of the wrist, without significant differences between the forearms with and without enthesopathy (Figure 2b). Immediately after the use of LC, the A_RMS_ value recorded during MVC increased to an average of 200–220 μV. Despite the significant increase in the A_RMS_ value after LC therapy, no difference was observed between the sides (ECR_LE_ vs. ECR_N/LE_) (Figure 2b). With respect to healthy muscles, an increase in the sEMG signal amplitude is interpreted by many researchers as an increase in the MU efficiency as it shows a greater number of motor units being involved in contraction or an increase in the frequency of bioelectrical discharges in the already working motor units [39,61,64]. This is confirmed by the studies showing the co-existence of an increase in this parameter with an increase in the isometric strength and endurance of quadriceps femoris muscle [76] and an increase in maximal isometric grip strength [77] following local stimulation with low temperature. The demonstrated increase in the value of amplitude for the contraction of the same muscle under the same conditions but following activation by cryogenic agents is evidence of the stimulating effect of LC on muscle excitation (bioelectrical activity), as evidenced by the presented research results (Figure 2b).

The source data on the effect of low temperatures on the sEMG signal amplitude are ambiguous. Both increases [51,78,79,80,81] and decreases [51,82] were reported, as well as no effect of cooling [53] on the value of amplitude after exposure to different cold temperatures. This discrepancy might be explained by different experimental protocols, including cooling procedures and measured muscle groups. As regards the literature data, results similar to our findings in the form of increased amplitude and decreased frequency have been obtained after the application of a 30-min cooling with an ice pack on the region of biceps brachii muscle [83] and foot plantar flexors [80], as well as previously cited reports, which showed an increase in the electromyographic parameters following the local stimulation with low temperature [77,84,85]. There are also reports in the literature that have contradicted the significant effect of cooling treatments on the bioelectric muscle activity [86,87]. The researchers reported that cryostimulation (temperature of −30 °C, 4 times, 3 days, following a physical exercise) did not significantly affect the electromyographic parameters being recorded during maximal isokinetic eccentric contractions of the elbow flexors [86]. Another study showed no significant effect of a single bout of whole-body cryotherapy (−110 °C, nitrogen) on the electromyographic parameters of the elbow flexors during isokinetic exercises [87]. However, after 20 min of cold-water immersion, a decrease in the amplitude of the MVC electromyogram was found (23 °C and 26 °C water temperature) [51]. The decrease in muscle excitation was probably related to the decrease in muscle temperature, which was the result of a long cooling (20 min). As mentioned above, muscle excitation is associated with the depolarisation of the sarcolemma following neural excitation, delivered to the muscle via the neuromuscular junction [40]. It has been shown that muscle cooling decreases the nerve conduction velocity [88]. In addition, based on the literature, it is known that a muscle temperature of 27 °C is assumed to be a critical temperature for initiating maximal isometric voluntary contraction [89,90]. Thus, it is likely that if the cooling procedure used causes the muscles to cool below the critical value for MVC performance, the MVC electromyogram amplitude value will decrease. The above will be the result of impaired recruitment of motor units for contraction, generating a lower total value of the bioelectric voltage recorded on the skin surface in the EMG test. On the other hand, a short time of stimulation by cryogenic temperature may cause an increase in the skin temperature due to vasodilation (cold-induced vasodilation, CIVD), which improves the efficiency of motor units and results in higher amplitude values recorded during contraction, as found in our study and parts of other studies [78,79,80,84,89] (Figure 2b). Given the wide variety of LC protocols used in the literature (exposure time, temperature, and state of matter of cooling agents), it is not surprising that there are conflicting study results, as noted in our previous paper [91]. Therefore, it should be emphasised that our study results should be related only to the procedure we used, i.e., a 3-min local exposure to liquid nitrogen vapours. Based on our own previously published research using the same cooling procedure, we know that the skin temperature quickly returns to the state before stimulation. Significant tissue (skin) surface cooling persists for up to 15 min after exposure to liquid nitrogen vapours [91]. Therefore, it seems that despite the extremely low temperature of the refrigerant used, due to the short time of its action (3 min), it was not able to cool the muscle to the critical 27 °C and limit its activation to contraction. The observed increase in the RMS amplitude proves the stimulating effect of the applied LC procedure on the state of muscle excitation (Figure 2b).

Based on the sEMG examination, the effect of local cryotherapy on neuromuscular functions related to fatigue has been assessed. The sEMG method is often used to assess muscle fatigue in rehabilitation (in people with various musculoskeletal dysfunctions) and sports (e.g., for training and assessing the risk of injury) and was also used in a few studies to assess muscle fatigue after exposure to low temperatures [47,51,78,92]. To our knowledge, there are no source data assessing the effect of LC on electromyographic indicators of muscle fatigue in patients with lateral epicondylitis enthesopathy. Myoelectric manifestations of muscle fatigue are quantified by monitoring the time course of the spectral variables (MNF, MDF) [41,42]. In the presented study, as expected, during a 60 s isometric contraction, the MNF value decreased, and the A_RMS_ value increased. This was found both on the side with and without LE and before and after the application of LC (Figure 3 and Figure 4). Throughout the 60 s observation time, A_RMS_ was significantly higher on the non-LE side versus the LE side (*p* < 0.0001) (Figure 3, Table 1). This is indicative of the stimulating effect of LC on muscle excitation. Elevated muscle excitation is maintained throughout a 60 s static contraction regardless of the presence of enthesopathy. Although cryotherapy increased the A_RMS_ value, its value remained lower throughout a 60 s contraction in relation to the ECR without enthesopathy (*p* < 0.0001) (Figure 3, Table 1). This explanation seems to be additionally confirmed by the reduced mean value of the MNF fatigue signal on the enthesopathy side (*p* < 0.0001) (Figure 4, Table 2).

The mechanism that is responsible for the above-mentioned physiological changes in the muscle activity is directly affected by reducing the skin and subcutaneous tissue temperature and, indirectly, by the muscle temperature (due to vasoconstriction, which still persists for about 1 min after treatment). This decelerates the nerve conduction velocity and reduces the reactivity of peripheral sensory-motor endings, as well as decreases the frequency of afferent information being provided by the muscle spindles in muscles [93]. The decreased nerve conduction velocity is considered an explanation for the decrease in mean signal frequency being observed [51,94,95,96,97]. Probably, the stimulation with low temperatures also decreases the number of active motor units, which would explain the decreased frequency that occurred after treatment, also reported in our study (Figure 4).

During fatigue isometric contraction, the sEMG frequency power spectrum is expected to shift to lower values. At the same time, the amplitude of the signal increases. This phenomenon is well established for static contractions at constant load levels and is believed to reflect the slowing of motor-unit action potentials, reduced conduction velocity and the synchronisation of motor units during fatigue [41,47]. Although the LC procedure clearly increased the amplitude value and decreased the MNF value, the regression lines marking the rate of these changes during isometric fatigue contractions did not differ significantly. Thus, the LC procedure did not affect the acceleration or deceleration of the muscle fatigue assessed using the surface electromyography method (Figure 3 and Figure 4).

The authors are aware of the limitation of the presented research. First, the thickness of the skin-fat fold was not assessed at the site of LC application and sEMG signal recording. Additional skinfold measurements would have been useful owing to the effect of the amount of local adipose tissue on the decrease in tissue temperature and modification of the sEMG signal. On the other hand, we assessed the dorsal area of the forearm, where both the thickness and the variability in the thickness of the adipose tissue are small. This does not change the fact that we do not recommend our results to directly relate to other areas with much greater adipose tissue thickness. Moreover, no skin or muscle temperatures were assessed before and after LC. Such temperature measurements may have provided useful information for the interpretation of the data. The fact that there is no female group may also be a limitation in the research. Therefore, the results of our study should be referred only to groups of men. Further research in this area could be enriched with a group of women and follow-up observation. In the described studies, however, the main goal was to understand the mechanism of an immediate, direct response to the LC.

Nevertheless, we believe that our research could be interesting, and the results are clinically useful. Enthesopathies are a common cause of musculoskeletal pain, and local cryotherapy is recommended as a supplement to the comprehensive treatment of these disorders. The local cryotherapy procedure used in this study is often applied in rehabilitation and sports. Therefore, we believe that the results of the present study might be of particular interest to physiotherapists and athletes’ trainers who use local cooling before exercise.

## 5. Conclusions

Local cryotherapy soothes the clinical symptoms of male office workers with lateral epicondylitis, causing immediate relief after exposure in the form of pain reduction during resisted wrist extension and increased pain-free grip. Since the increase in the strength of the pain-free grip occurred only on the LE side, we believe that it is caused by the analgesic effect of muscle cooling. A 3-min exposure to liquid nitrogen vapours also causes increased muscle excitation expressed by an increase in the value of ARMS. The increases in muscle excitation occur during rest, maximum voluntary contraction, and isometric fatigue contraction on both the healthy side and the side affected by LE. At the same time, LC does not affect muscle fatigue, as shown by the approximate values of the linear regression coefficients characterising the rate of the increase in the ARMS value and the decrease in the MNF value during 60 s contractions.

## Figures and Tables

**Table 1:** Analysis of the effect of local cryotherapy on the A_RMS_ value recording during a 60 s contraction.

Median (Min.–Max.)	T_0_ [µV]	T_1_	Wilcoxon Test
**ECR _N/LE_**	192.62 (169.07–242.17)	250.33 (223.99–278.63)	Z = 6.735 *p* < 0.0001
**ECR _E_**	121.21 (101.73–159.56)	179.27 (156.42–202.77)	Z = 6.165 *p* < 0.0001
**U Manna-Whitneya Test**	Z = −9.261 *p* < 0.0001	Z = −9.441 *p* < 0.0001	

ECR—extensor carpi radialis longus and brevis muscles, _LE_—lateral enthesopathy, _N/LE_—healthy side, T_0_—before local cryotherapy, T_1_—after local cryotherapy.

**Table 2:** Analysis of the effect of local cryotherapy on the MNF value recording during a 60 s contraction.

Median (Min.–Max.)	T_0_	T_1_	Wilcoxon Test	
**ECR _N/E_**	80.51818 (73.07–91.13)	69.41932857 (64.32–79.31)	Z = 6.736	*p* < 0.0001
**ECR _E_**	73.32914 (66.17–81.62)	65.4 (59.47–74.46)	Z = 6.738	*p* < 0.0001
**Mann–** **Whitney U Test**	Z = 6.836 *p* < 0.0001	Z = 5.393 *p* < 0.0001		

ECR—extensor carpi radialis longus and brevis muscles, _LE_—lateral enthesopathy, _N/LE_—healthy side, T_0_—before local cryotherapy, T_1_—after local cryotherapy.
